# Ballistocardiogram suppression in concurrent EEG‐MRI by dynamic modeling of heartbeats

**DOI:** 10.1002/hbm.25965

**Published:** 2022-06-13

**Authors:** Hsin‐Ju Lee, Simon J. Graham, Wen‐Jui Kuo, Fa‐Hsuan Lin

**Affiliations:** ^1^ Physical Sciences Platform Sunnybrook Research Institute Toronto Ontario Canada; ^2^ Department of Medical Biophysics University of Toronto Toronto Ontario Canada; ^3^ Institute of Neuroscience National Yang Ming Chiao‐Tung University Taipei Taiwan; ^4^ Brain Research Center National Yang‐Ming Chiao‐Tung University Taipei Taiwan

**Keywords:** artifact, cardiac, dynamic modeling, EEG, MRI, physiological noise, pulse

## Abstract

The ballistocardiogram (BCG), the induced electric potentials by the head motion originating from heartbeats, is a prominent source of noise in electroencephalography (EEG) data during magnetic resonance imaging (MRI). Although methods have been proposed to suppress the BCG artifact, more work considering the variability of cardiac cycles and head motion across time and subjects is needed to provide highly robust correction. Here, a method called “dynamic modeling of heartbeats” (DMH) is proposed to reduce BCG artifacts in EEG data recorded inside an MRI system. The DMH method models BCG artifacts by combining EEG points at time instants with similar dynamics. The modeled BCG artifact is then subtracted from the EEG recording to suppress the BCG artifact. Performance of DMH was tested and specifically compared with the Optimal Basis Set (OBS) method on EEG data recorded inside a 3T MRI system with either no MRI acquisition (Inside‐MRI), echo‐planar imaging (EPI‐EEG), or fast MRI acquisition using simultaneous multi‐slice and inverse imaging methods (SMS‐InI‐EEG). In a steady‐state visual evoked response (SSVEP) paradigm, the 15‐Hz oscillatory neuronal activity at the visual cortex after DMH processing was about 130% of that achieved by OBS processing for Inside‐MRI, SMS‐InI‐EEG, and EPI‐EEG conditions. The DMH method is computationally efficient for suppressing BCG artifacts and in the future may help to improve the quality of EEG data recorded in high‐field MRI systems for neuroscientific and clinical applications.

## Graphical abstract

The ballistocardiogram (BCG), defined as the induced electric potentials by the head motion originating from heartbeats, is a prominent source of noise in electroencephalography (EEG) data during magnetic resonance imaging (MRI). Here, a method called “dynamic modeling of heartbeats” (DMH) is proposed to reduce BCG artifacts in EEG data recorded inside an MRI system. The DMH method models BCG artifacts by pooling EEG signals at time instants with similar cardiac dynamics. The modeled BCG artifact is then subtracted from the EEG recording to suppress the BCG artifact.

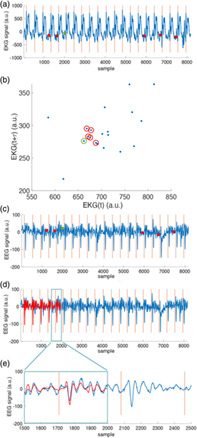

## 1 INTRODUCTION

Electroencephalography (EEG) recording and functional magnetic resonance imaging (fMRI) in the same setting (Ives et al., 1993) allows for the characterization of neuronal and hemodynamic signals. This technique has been fruitful in elucidating neurovascular coupling effects (Debener et al., 2005; Laufs et al., 2003) and localizing epileptiform activity generators (Gotman et al., 2004; Ives et al., 1993; Krakow et al., 1999; Seeck et al., 1998). However, EEG recorded concurrently with magnetic resonance imaging (MRI) is seriously deteriorated by the induced voltage caused by the switching of MRI gradient coils. Gradient artifacts in EEG data can be effectively suppressed by artifact template estimation and subtraction (Allen et al., 2000), blind source separation, frequency filtering, dictionary learning (Bullock et al., 2021), or by minimizing the duty cycles of MRI acquisition (Lee et al., 2020).

Ballistocardiogram (BCG) artifacts also occur when EEG data are collected inside an MRI system. In the present context, the BCG artifact is the EEG signal fluctuation that is obtained within a strong static magnetic field, when scalp electrodes move due to cardiac pulsatility (Allen et al., 1998) or caused by the Hall effect of the pulsatile blood flow (Muri et al., 1998). The amplitude of the BCG artifact ranges between 150 and 200 μV, which is larger than the typical electrophysiological activity of interest (approximately 1–10 μV; Allen et al., 1998; Huster et al., 2012; Mulert & Lemieux, 2009). The duration of the BCG artifact typically lasts for approximately 1 s (Niazy et al., 2005a). Due to its high amplitude, significant spectrotemporal overlap with neurophysiologic signals, and large variations in the waveform shape, timing, and intensity across time and electrode locations, BCG artifacts substantially reduce the sensitivity and specificity for detecting the EEG signals of interest. The amplitude of the artifact scales with the field strength of MRI (Debener et al., 2008; Mullinger, Havenhand, & Bowtell, 2013). Therefore, suppressing BCG artifacts becomes more pressing when EEG data are recorded inside a high‐field (≥3 T) MRI system.

Among methods to suppress BCG artifacts, several estimate an artifact “template” waveform and then subtract the template from the contaminated EEG signals (Allen et al., 1998;Ellingson et al., 2004; Sijbersa et al., 2000). The estimation of an artifact template is based on the collection of EEG signals temporally aligned to a specific time point in the cardiac cycle, such as the peak of the QRS complex. Either the average (Allen et al., 1998) or a combination of aligned EEG signal segments (Ellingson et al., 2004; Sijbersa et al., 2000) are taken as the artifact template. Although widely used, these methods do not consider the variations in the electrocardiogram (EKG) shape, amplitude, and QRS complex timing, which may lead to out‐of‐phase artifact subtraction, systematic errors, and large residuals (Jorge et al., 2019; Musso et al., 2011; Niazy et al., 2005b).

Methods based on Principal Component Analysis (Niazy et al., 2005b) and Independent Component Analysis (Benar et al., 2003; Nakamura et al., 2006; Srivastava et al., 2005) provide alternative ways to suppress BCG artifacts. However, these methods require a sufficient number of sensors to allow for the decomposition of the measurements into “signal” and “noise” components. Categorizing the decomposed components as either “signal” or “noise” typically requires a heuristic definition, which may reduce the performance of BCG artifact suppression (Grouiller et al., 2007; Nakamura et al., 2006). More recently, a reference‐free harmonic regression modeling approach was proposed to suppress BCG artifacts (Krishnaswamy et al., 2016). This method shows comparable performance with the Optimal Basis Set (OBS) method (Niazy et al., 2005b) in revealing evoked potentials, while suppressing BCG artifacts (21% in harmonic regression vs. 26% in OBS) (Krishnaswamy et al., 2016).

Motivated by a causal modeling method (Sugihara et al., 2012), the present work proposes a new method called “dynamic modeling of heartbeats” (DMH) to suppress BCG artifacts. The DMH method estimates the instantaneous EEG signals at specific phases in the cardiac cycle by combining EEG signals at those phases in other cardiac cycles showing similar dynamic features, which are EKG signals at different latencies within a cardiac cycle. The resulting modeled EEG signal is taken as the estimated BCG artifact and subtracted from the original recording to generate artifact‐suppressed EEG signals. Methodology and empirical results are presented of using DMH to suppress BCG artifact in EEG evoked potentials recorded concurrently with MRI. Specifically, the DMH performance in identifying evoked potentials is benchmarked with respect to the OBS method. Substantial improvement in BCG artifact suppression will motivate the application of DMH to neuroscience and clinical applications of concurrent EEG‐MRI experiments, to obtain high‐quality concurrent measurements of electrophysiological and hemodynamic responses, particularly in cases where the MRI gradient artifact over EEG is effectively suppressed.

## 2 METHODS

### 2.1 Participants

This study was approved by the Research Ethics Board of Sunnybrook Research Institute and Institutional Review Board of National Yang Ming Chiao Tung University. Thirty healthy control participants (age: 21–45 years; 18 female) gave written free and informed consent before participating in the study. Nine participated in the evoked response experiment. Part of the visual evoked response data was analyzed in a previous study of fast MRI and EEG conducted in the laboratory (Lee et al., 2020). The data that support the findings of this study are available on request from the corresponding author. The data are not publicly available due to privacy or ethical restrictions.

### 2.2 Experiment design

The experiments involved recording evoked EEG responses in various MRI acquisition environments. The measurement of evoked responses involved steady‐state visual evoked potentials (SSVEPs). Specifically, participants were instructed to fixate visually on a crosshair at the center of the display screen. To ensure that participants maintained visual fixation, they were instructed to press a button when the crosshair changed color from black to red. The red crosshair appeared for 1 s randomly throughout the experiment. Asynchronous with the crosshair stimulus, flashing checkerboard patterns (flashing frequency = 7.5 Hz, stimulus duration 1 s) were also presented randomly with a minimal inter‐stimulus interval of 2 s to elicit SSVEPs. The checkerboard subtended 4.3° of visual angle and contained 24 evenly distributed radial wedges with eight concentric rings of equal width. The 7.5‐Hz flashing checkerboard stimuli were expected to elicit a strong SSVEP with a frequency of 15 Hz. Onsets of checkerboard flashing were temporally jittered between 0.2 and 0.9 s after the beginning of each MRI acquisition of the brain volume to minimize artifacts caused by MRI gradient coil switching in concurrent fast fMRI and EEG acquisition (see details below). Three six‐minute runs were collected for each condition from each participant.

### 2.3 MRI collection

All MRI data were measured on a 3 T MRI scanner using a 64‐channel head–neck receiver coil array (Prisma or Skyra, Siemens, Erlangen, Germany) with a hole at the vertex of the head coil for routing the EEG cables. Structural images were acquired with the magnetization‐prepared rapid gradient echo (MPRAGE) pulse sequence (repetition time TR = 2530 ms, echo time TE = 3.03 ms, isotropic voxel resolution = 1 mm, field of view FOV = 256 mm, flip angle = 7°, matrix size = 224 × 256, generalized auto‐calibrating partial parallel acquisition [GRAPPA] acceleration factor = 2). As EEG recorded inside MRI suffers from artifacts caused by MRI gradient coil switching and heartbeats, we took EEG with two different kinds of fMRI to probe the performance of BCG artifact suppression with low and high levels of residual gradient artifacts. Specifically, functional images were acquired with a fast fMRI sequence (simultaneous multi‐slice inverse imaging (SMS‐InI; Hsu et al., 2017); TR/TE = 2000/30 ms, FOV = 210 mm, flip angle = 30°, resolution = 5 × 5 × 5 mm^3^, slice numbers = 24). Spatial encoding was performed in 0.1 s, leaving 1.9 s (95% of the TR interval) free from MRI gradient coil operation. We previously showed that concurrent temporally sparse fast fMRI and EEG gave much reduced gradient artifact (Lee et al., 2020). For comparison, *T*_2_*‐weighted echo‐planar imaging (EPI) was also acquired for fMRI with a typical spatiotemporal resolution (TR/TE = 2,000/36 ms, FOV = 224 mm, flip angle = 90°, number of slices = 30, resolution = 3.5 × 3.5 × 4 mm, GRAPPA acceleration = 2).

### 2.4 EEG data collection

EEG was recorded inside the 3 T MRI scanner using an MRI‐compatible system (BrainAmp MR Plus, Brain Products, Gilching, Germany) with a 32‐channel EEG cap (BrainCap MR, Brain Products, Gilching, Germany). Electrodes were arranged following the 10–20 international standard. The EEG data were referenced with respect to the FCz electrode, with the ground reference taken at the AFz electrode. The EKG was also measured by placing an electrode on the back of the participant.

To ensure highly synchronous EEG and EKG recordings with respect to the fMRI acquisitions, an established procedure (Mandelkow et al., 2006) was adopted using a frequency divider and phase‐locking device as part of the EEG system (BrainAmp MR Plus, Brain Products, Gilching, Germany). The phase‐locking device received the 10 MHz transistor‐to‐transistor logic (TTL) signal from the clock board of the MRI system via a coaxial cable and produced a 5‐kHz output signal to synchronize the EEG acquisition. The MRI TR value recorded by the EEG system was confirmed to match the prescribed TR value at the MRI console with 5‐kHz sampling rate. The impedance of each electrode was verified as <9 kΩ (including the built‐in 5 kΩ impedance) after applying the conductive gel. The EEG cap wire bundle was straightened and fixed along the main magnetic field for 50 cm and connected to an EEG amplifier at the rear of the magnet (just outside the bore) to reduce artifacts generated by the wire (Mullinger, Castellone, & Bowtell, 2013). The positions of electrodes over the scalp of a participant were measured by a digitizer (Fastrak, Polhemus, Vermont, Canada). These positions were used to register EEG electrodes with the head model derived from structural MRI.

EEG was measured separately in three different conditions. EPI‐EEG and SMS‐InI‐EEG denote the concurrent recording of EEG with EPI and SMS‐InI, respectively. In addition, EEG was recorded inside the MRI system without imaging (Inside‐MRI) providing a condition that yielded EEG signals with BCG artifact but no gradient artifact (GA).

### 2.5 EEG data analysis

#### 2.5.1 GA suppression and filtering

The EEG processing was implemented in MATLAB (Mathworks, Natick, MA, U.S.A.). For EPI‐EEG and SMS‐InI‐EEG, GA was suppressed using the average artifact subtraction (AAS) method (Allen et al., 2000). To account for the timing difference in the clock accuracy between MRI (10 MHz) and EEG (5 kHz) systems, further alignment between the GA template and the EEG data was achieved by interpolating with an accuracy of 0.2, 0.02, 0.002, and 0.0002 samples in four iterations to achieve numerical sampling rates of 0.025, 0.25, 2.5, and 25 MHz, respectively. The GA template was dynamically estimated over seven TR intervals. The EEG data were further zero‐phase band‐pass filtered between 1 and 50 Hz, and down‐sampled to 500 Hz.

#### 2.5.2 BCG artifact suppression

To suppress BCG artifacts, peaks of the QRS complex were first identified automatically from the recorded EKG waveform (Pan & Tompkins, 1985). The subsequent proposed approach was to build a dynamic model of the EEG signals with BCG artifacts. This procedure was motivated by the convergent cross mapping method (Sugihara et al., 2012), a method to test causal relationship between two time series. Instead of estimating a cause‐and‐effect relationship, the method was modified to estimate the BCG artifact templates. The modeled EEG time series was then subtracted from the original one to yield the signal with suppressed BCG artifacts.

Describing the DMH method in mathematical detail, given an EKG time series *s*_EKG_(*t*) with the temporal index *t*, instants were first detected corresponding to the peak timing of the QRS complex and these were taken as the temporal references for individual cardiac cycles. Next, the mapping *f*(*t*) was established to transform the temporal index *t* to the *m* ^th^ cardiac cycle with a latency *φ*:

(1) $$f\left(t\right)\to \left\{m,\varphi \right\},m=1,\cdots ,m_{\max}$$

Thus, *s*_EKG_(*t*) was mapped to its cardiac cycle *m* and latency φ according to

(2) $${s_{\mathrm{EKG}}\left(t\right)\to s}_{\mathrm{EKG}}^{m}\left(\varphi \right),$$

where $s_{\mathrm{EKG}}^{m}\left(\varphi \right)$ represents the EKG signal at latency φ with respect to the *m*th QRS complex in the recording.

In analogous fashion, the mapping *f*(t) also allowed a given EEG signal at the temporal index *t*, $s_{\mathrm{EEG}}\left(t\right)$, to be related to the *m*th cardiac cycle with a latency φ.

(3) $$s_{\mathrm{EEG}}\left(t\right)\to s_{\mathrm{EEG}}^{m}\left(\varphi \right).$$

Note that φ in the *m*th EKG cycle can be related to the beginning of the recording by *f*(*t*).

An *e*‐dimensional manifold of cardiac dynamics was created from time‐lagged samples at multiples of latency τ of the EKG:

(4) $$k_{\mathrm{EKG}}^{m}\left(\varphi \right)=\left[s_{\mathrm{EKG}}^{m}\left(\varphi \right),s_{\mathrm{EKG}}^{m}\left(\varphi +\tau \right),\cdots ,s_{\mathrm{EKG}}^{m}\left(\varphi +\left(e-1\right)\tau \right)\right],$$

where φ denotes the latency within the *m*th cardiac cycle with respect to its QRS complex. e denotes the dimension of cardiac dynamics manifold, that is, the number of data points within a cardiac cycle to describe the dynamic feature at latency φ in the *m*th cardiac cycle. As so, $k_{\mathrm{EKG}}^{m}\left(\varphi \right)$ is a vector of e EKG values sampled in multiples of 𝜏 with a latency φ with respect to the QRS complex in the *m*th cardiac cycle. Next, a matrix **K**_EKG_ $\left(\varphi \right)$ was created by vertically concatenating $k_{\mathrm{EKG}}^{m}\left(\varphi \right)\;$across cardiac cycles:

(5) $$K_{\mathrm{EKG}}\left(\varphi \right)={\left[k_{\mathrm{EKG}}^{1}\left(\varphi \right);k_{\mathrm{EKG}}^{2}\left(\varphi \right);\ldots ;k_{\mathrm{EKG}}^{m_{\max}}\left(\varphi \right)\right]}^{T},$$

where the superscript T denotes the matrix transpose. For the EKG at cycle *m*, it was then possible to search across cardiac cycles to identify *n* cycles that were in the proximity of $k_{\mathrm{EKG}}^{m}\left(\varphi \right)$ in the **K**_EKG_ $\left(\varphi \right)$ manifold. The “neighboring” cardiac cycles were indexed by *m*_i_, *i* = 1, …, *n;* $n\leq m_{\max}$. In the **K**_EKG_ $\left(\varphi \right)$ manifold, the distance $d^{m_{i}}$ between $k_{\mathrm{EKG}}^{m}\left(\varphi \right)$ and $k_{\mathrm{EKG}}^{m_{i}}\left(\varphi \right)$ was measured by the Euclidean distance. All *n* cardiac cycles in the vicinity of the *m* ^th^ cardiac cycle were then sorted in the ascending order: $d^{m_{1}}\leq d^{m_{2}}\leq \cdots \leq d^{m_{n}}$. A linear combination of EEG signals in all cardiac cycles (*i* = 1, …, *n; n* $\leq m_{\max}$) with the latency φ was then used to develop a model of the EEG signal, ${\hat{s}}_{\mathrm{EEG}}^{m}\left(\varphi \right)$, in the *m* ^th^ cardiac cycle with the latency φ, as follows:

(6) $${\hat{s}}_{\mathrm{EEG}}^{m}\left(\varphi \right)=\sum_{i=1}^{n}w_{i}s_{\mathrm{EEG}}^{m_{i}}\left(\varphi \right).$$

Suggested by the prediction procedure in modeling dynamics in a manifold (Sugihara et al., 2012), the weights $w_{i}$ were calculated by

(7) $$u_{i}=e^{-d^{m_{i}}}/e^{-d^{m_{1}}},$$

(8) $$w_{i}=u_{i}/\sum_{i=1}^{n}u_{i}.$$

Briefly, the weighting coefficients $w_{i}\;$ and data $s_{\mathrm{EEG}}^{m_{i}}\left(\varphi \right)$ to model the EEG measurement $s_{\mathrm{EEG}}^{m}\left(\varphi \right)$ by ${\hat{s}}_{\mathrm{EEG}}^{m}\left(\varphi \right)$ were based on the similarity between the representations of $s_{\mathrm{EEG}}^{m}\left(\varphi \right)$ and $s_{\mathrm{EEG}}^{m_{i}}\left(\varphi \right)$ in the cardiac dynamics in **K**_EKG_ $\left(\varphi \right)$. Although alternative interpolation methods may also work in DMH, for the sake of computational simplicity, we only limited ourselves to a linear interpolation method to model $s_{\mathrm{EEG}}^{m}\left(\varphi \right)$ by ${\hat{s}}_{\mathrm{EEG}}^{m}\left(\varphi \right)$. The modeled EEG ${\hat{s}}_{\mathrm{EEG}}^{m}\left(\varphi \right)$ can be transformed to the temporal index *t* by a mapping

(9) $${\hat{s}}_{\mathrm{EEG}}^{m}\left(\varphi \right)\to {\hat{s}}_{\mathrm{EEG}}\left(t\right).$$

Because ${\hat{s}}_{\mathrm{EEG}}\left(t\right)$ was linearly interpolated from the EEG recording with the same cardiac latency in other cardiac cycles, ideally it contained only BCG features without other physiological information specific to time instant *t*. If the effect of interest occurred asynchronously to cardiac cycles, then subtracting ${\hat{s}}_{\mathrm{EEG}}\left(t\right)$ from $s_{\mathrm{EEG}}\left(t\right)$ yielded $s_{\mathrm{EEG}}^{\mathrm{BCG}}\left(t\right)$ as the EEG signal after BCG artifact suppression:

(10) $$s_{\mathrm{EEG}}^{\mathrm{BCG}}\left(t\right)=s_{\mathrm{EEG}}\left(t\right)-{\hat{s}}_{\mathrm{EEG}}\left(t\right).$$

**FIGURE 1:**
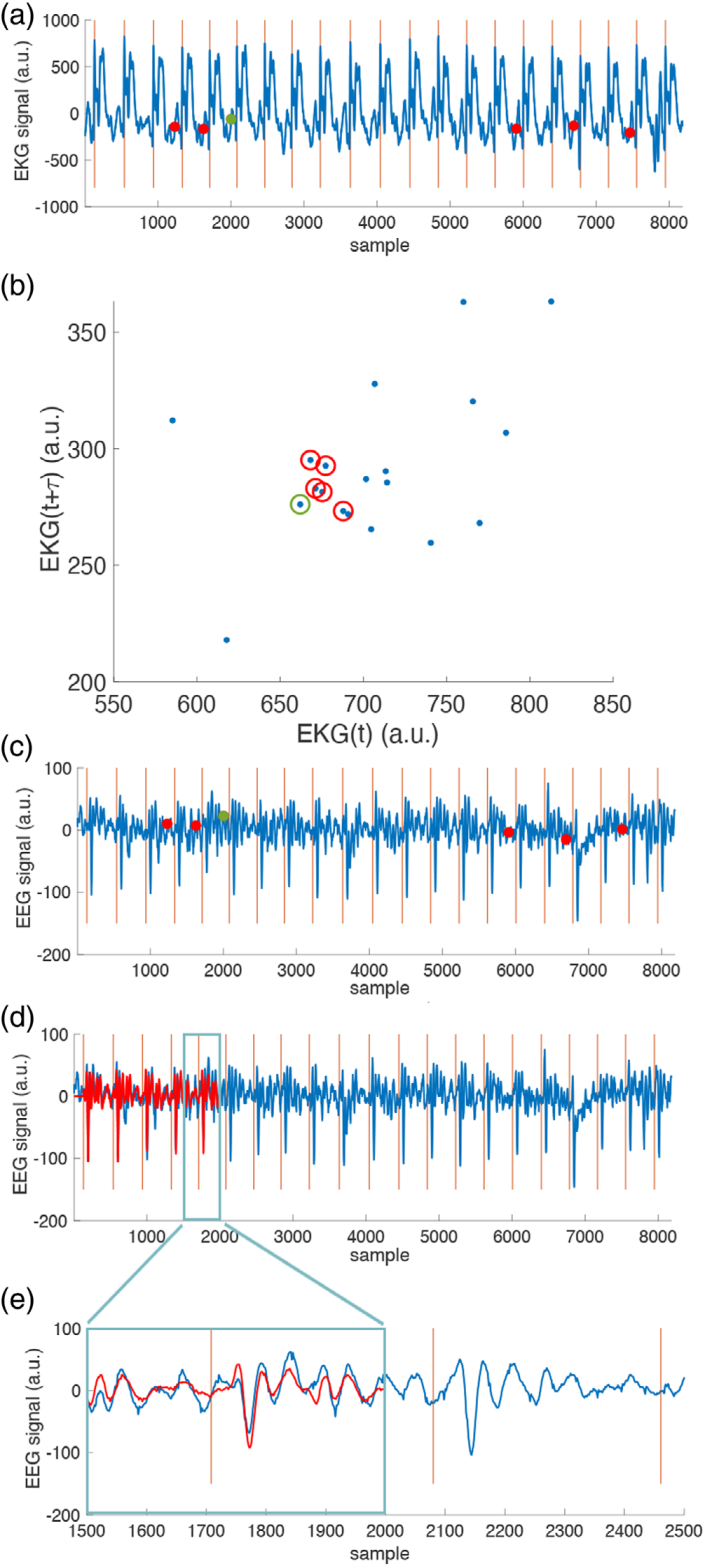
An illustration of the dynamic modeling of heartbeats approach to suppress the BCG artifacts in EEG. (a) An EKG waveform. (b) a two‐dimensional manifold (*e* = 2) of cardiac dynamics. Each blue dot indicates one cardiac cycle. A pair of EKG signals at its QRS peak and a delay *τ* = 10 samples was used to represent one cardiac cycle. The dot with a green circle indicates the cardiac cycle of specific interest. five nearest cardiac cycles on this 2D manifold are indicated by red circles. (c) EEG waveforms. red vertical lines indicate the detected QRS peaks. The green and red dots correspond to the analogous cardiac cycles identified on the 2D manifold (a)). (d) the recorded (blue) and interpolated (red) EEG waveforms based on the EEG signals at five instants, whose cardiac cycles are nearest to the current one on the 2D manifold. Both waveforms are expected to have similar BCG artifacts. (e) Details of the recorded (blue; $s_{\mathrm{EEG}}\left(t\right)$) and interpolated (red; ${\hat{s}}_{\mathrm{EEG}}\left(t\right)$) EEG waveforms showing reasonable estimates of the EEG signal with clear BCG artifacts

Figure 1 illustrates the procedure of building a two‐dimensional K_EKG_ (*e* = 2) and seeking five nearest neighbors (*n* = 5) to derive ${\hat{s}}_{\mathrm{EEG}}\left(t\right)$. In this example, the time instant of interest (green dot) has five time instants (red dots) in the EKG waveform (Figure 1a) with the same cardiac phase and the most similar dynamics features, which are EKG signals at two latencies (0 and τ=10 samples) within the cardiac cycle (Figure 1b). EEG signals at the interested time instant (red dot) and five time instants of the most similar dynamic features in terms of the Euclidean distance in the two‐dimensional manifold are shown in Figure 1c. The modeled (red) and acquired (blue) EEG signals with the BCG artifact ${\hat{s}}_{\mathrm{EEG}}\left(t\right)$ are shown in Figure 1d and e (temporally zoomed).

For comparison, BCG artifacts in the EEG data were separately suppressed by the optimal basis set (OBS) method (Niazy et al., 2005a). Suggested by previous studies on OBS, the order of the Principal Component Analysis was chosen to be three (Debener et al., 2007; Niazy et al., 2005b).

Subsequently, the SSVEPs were calculated by first extracting EEG signals between 200 ms before and 1000 ms after the onset of each visual stimulus for all trials of a given measurement (EPI‐EEG, SMS‐InI‐EEG, and inside‐MRI). For all EEG trials, the constant and the linear drift were removed by linear regression. Spurious trials with a maximum EEG signal >700 μV were excluded. The SSVEPs were then derived by averaging across trials at each electrode. Oscillatory features in the evoked EEG signals were quantified using the Morlet wavelet transform with a temporal window of 5 cycles. The 15‐Hz oscillations were then investigated for the SSVEPs.

#### 2.5.3 EEG source modeling

The SSVEP measured by EEG electrodes at scalp originated from the visual cortex. It is difficult to define the ensemble of EEG electrodes and their relative contribution to summarize the SSVEP over the scalp. In order to provide spatially specific assessment of SSVEP, we estimated the distribution of neuronal activity responsible for generating SSVEP. The scalp EEG data and the neuronal current sources at time *t* were related to each other by the linear equation

(11) $$y\left(t\right)=A\;x\left(t\right)+n\left(t\right),$$

where **y**(*t*) denoted the collection of EEG data across electrode contacts, **x**(*t*) denoted the neuronal current strength, and **n**(*t*) denoted the contaminating noise, and **A** was the lead field matrix (Lin et al., 2006). Specifically, for a unit current dipole source at location r in the +*x*, +*y*, or + *z* direction, the electric potentials measured at all electrode contacts were

(12) $$a\left(r\right)=\left[a_{x}\left(r\right),a_{y}\left(r\right),a_{z}\left(r\right)\right].$$

Assembling **a**(**r**) across all possible current dipole source locations created the lead field matrix **A**:

(13) $$A=\left[a\left(r_{1}\right),a\left(r_{2}\right),\ldots ,a\left(r_{k}\right),\ldots \right],k=1,\ldots ,d.$$

where *d* denotes the total number of current dipole source locations and **r**_*k*_ denotes the location of the *k* ^th^ current dipole source location.

The lead field matrix **A** was created from the *T*_*1*_‐weighted MPRAGE MRI data using scalp, skull, and brain models generated by FreeSurfer (https://surfer.nmr.mgh.harvard.edu). Potential EEG source locations at the gray and white matter boundary were identified with approximately 5‐mm separation between the nearest neighboring source locations. The locations of EEG electrodes were manually registered to the scalp model. The matrix **A** was then calculated by the *OpenMEEG* package (Gamfort et al., 2010) (https://openmeeg.github.io/).

The Minimum‐Norm Estimate (MNE) was used to estimate x(*t*) (Hämäläinen & Ilmoniemi, 1984):

(14) $$x_{\mathrm{MNE}}\left(t\right)=RA^{T}{\left(\mathrm{AR}A^{T}+\lambda C\right)}^{-1}y\left(t\right),$$

where **R** was the source covariance matrix and **C** was the noise covariance matrix

(15) $$C=\left\langle n\left(t\right)n^{T}\left(t\right)\right\rangle ,$$

with the operator $\left\langle ∙\right\rangle$ taking the ensemble average across realizations. In practice, **C** was estimated from **y**(*t*) during the pre‐stimulus interval (from −200 to 0 ms) with data concatenated across trials in the SSVEP measurements. The regularization λ tuned the balance between the strength of the estimated neural current strength and the discrepancy between the modeled and measured data. The value λ=10 was used in this study as suggested by previous work (Lin et al., 2006).

The spatial distribution of estimated neuronal currents at each time instant from each participant was then spatially registered to an arbitrarily selected individual: “*fsaverage*” in the FreeSurfer library. This registration was done via a spherical coordinate system (Fischl et al., 1999). The neuronal currents were averaged across participants for each condition separately. The significance of the neuronal current distribution was estimated at each source location by calculating the ratio between the instantaneous value and the standard deviation calculated over the baseline interval. These ratios constituted dynamic statistical parametric maps (dSPMs) and were reported to follow a *t*‐like distribution (Dale et al., 2000). To model the oscillatory neuronal current distributions in the brain, EEG signals were first filtered by the Morlet wavelet transform and then modeled by the MNE. The filtered coefficients were then used to generate the MNE and dSPMs.

### 2.6 Performance measures

The performance of BCG artifact suppression was measured for the SSVEP experiments by first characterizing the difference of 15‐Hz oscillatory responses, for scalp EEG at O1, O2, and Oz electrodes over the interval of + 0.2 and 1.0 s after the visual stimulus onset. The electrode topology and time interval were chosen based on practice in previous SSVEP studies (Norcia et al., 2015; Srinivasan et al., 2006). The SSVEP waveforms were also characterized in terms of their total power, transient response, and oscillatory response. The detection of neuronal sources for SSVEPs was also compared between OBS and DMH methods for the three MRI conditions (Inside‐MRI, EPI‐EEG, SMS‐InI‐EEG) using Receiver Operating Characteristic curves (Hanley & McNeil, 1982), where the boundary of the primary visual cortex was delineated from an independent structural MRI and fMRI studies (Benson et al., 2014; Hinds et al., 2008). The true‐positive and false‐negative detection was taken as the areas intersecting the anatomically defined primary visual cortex and the brain area showing significant and insignificant 15‐Hz oscillations, respectively. The true‐negative and false‐positive detection was taken as the areas intersecting the brain area outside the anatomically defined primary visual cortex and the brain area showing insignificant and significant 15‐Hz oscillations, respectively. The difference in the detection of SSVEP between OBS and DMH was statistically quantified by a bootstrap analysis, where data across participants were sampled with replacement for 100 times to generate 100 bootstrap samples of the group response. A two‐sample *t*‐test was applied to evaluate if the difference between these bootstrap group responses of OBS and DMH was significant.

## 3 RESULTS

**FIGURE 2:**
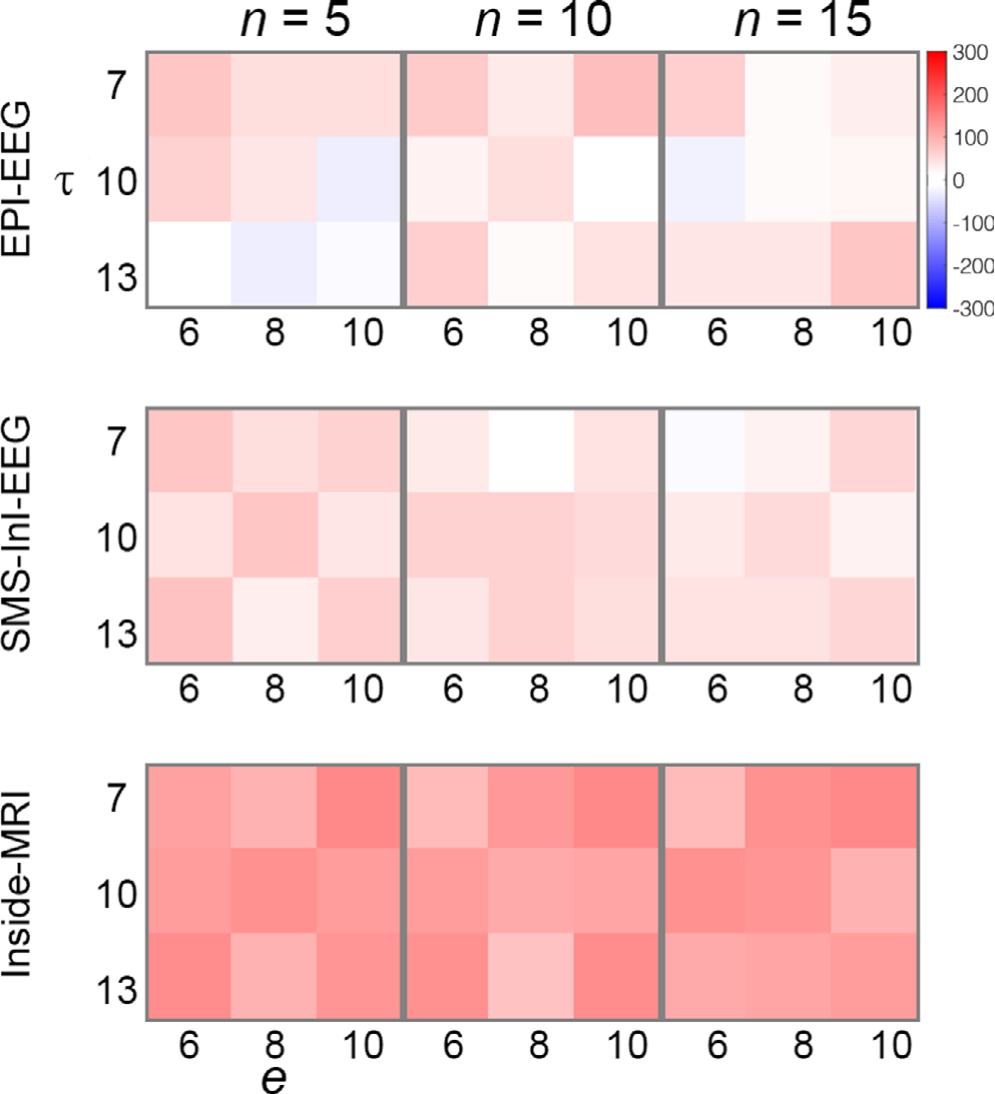
The difference of the evoked response 15‐Hz oscillatory power between DMH and OBS processing for different DMH parameter combinations. Differences were averaged across O1, O2, and Oz electrodes and participants. Positive differences correspond to larger 15‐Hz oscillatory power by DMH compared to OBS

Figure 2 shows the post‐stimulus 15‐Hz oscillatory power difference between SSVEP results using DMH and OBS to suppress BCG artifacts. These differences were calculated for different combinations of DMH parameters (*n* = 5, 10, or 15; *e* = 6, 8, or 10; τ = 7, 10, or 13), for EEG collected concurrently with EPI (EPI‐EEG), SMS‐InI (SMS‐InI‐EEG), and inside the magnet without MRI (Inside‐MRI). Except for a few cases in EPI‐EEG, DMH generally improved the detection of post‐stimulus 15‐Hz oscillation of the averaged EEG across O1, O2, and Oz electrodes by showing positive power differences for DMH compared to OBS regardless of MRI condition (EPI‐EEG, SMS‐InI‐EEG, or Inside‐MRI). This suggested that DMH outperformed OBS stably among parameter combinations. In the following sections, all results for DMH are provided with the parameter combination of (*ε* = 8, *τ* = 10, and *n* = 10) which reasonably approximate the average performance of the method.

**FIGURE 3:**
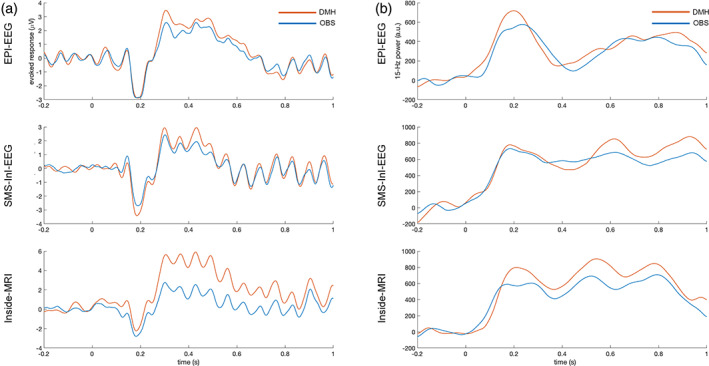
Average evoked responses (a) and average 15‐Hz time‐frequency representation amplitude (b) across O1, O2, and Oz electrodes

Figure 3a shows the averaged EEG traces across O1, O2, and Oz electrodes using OBS or DMH to suppress BCG artifacts. The averaged EEG power after the stimulus onset was found to be consistently higher after using DMH to suppress BCG artifacts in comparison to using OBS, regardless of whether EEG was recorded concurrently with EPI (EPI‐EEG; OBS: 1.73 +/− 0.03 μV^2^, DMH: 2.55 +/− 0.04 μV^2^; two‐sample test: *t* = 13.76, *p* < 10^−10^), SMS‐InI (SMS‐InI‐EEG; OBS: 1.08 +/− 0.02 μV^2^, DMH: 1.78 +/− 0.04 μV^2^; two‐sample test: *t* = 13.51, *p* < 10^−10^), or inside the magnet without MRI (Inside‐MRI; OBS: 1.51 +/− 0.03 μV^2^, DMH: 8.64 +/− 0.14 μV^2^; two‐sample test: *t* = 54.32, *p* < 10^−10^). This amounted to a 48% (2.55/1.73–1), 65% (1.78/1.08–1), and 472% (8.64/1.51–1) gain in the averaged EEG power for EPI‐EEG, SMS‐InI‐EEG, and Inside‐MRI, respectively.

Figure 3b shows the 15‐Hz time‐frequency representation (TFR) of the EEG waveform averaged across O1, O2, and Oz electrodes. For EPI‐EEG, the difference between waveforms after OBS and DMH suppressing BCG artifacts was not visually clear. This difference was clearer for SMS‐InI‐EEG after +500 ms. For Inside‐MRI, the 15‐Hz TFR was consistently stronger with DMH than with OBS after +200 ms. Two‐sample *t*‐tests were performed to quantify the TFR difference between OBS and DMH processing. All conditions show significantly larger 15‐Hz TFR by DMH than OBS (EPI‐EEG: OBS: 326.24 +/− 2.16, DMH: 378.38 +/− 2.27; *t* = 14.73, *p* < 10^−10^; SMS‐InI‐EEG: OBS: 566.64 +/− 2.16, DMH: 627.65 +/− 2.77; *t* = 17.38, *p* < 10^−10^; Inside‐MRI: OBS: 497.94 +/− 2.50, DMH: 606.68 +/− 3.47; *t* = 25.41, *p* < 10^−10^).

**FIGURE 4:**
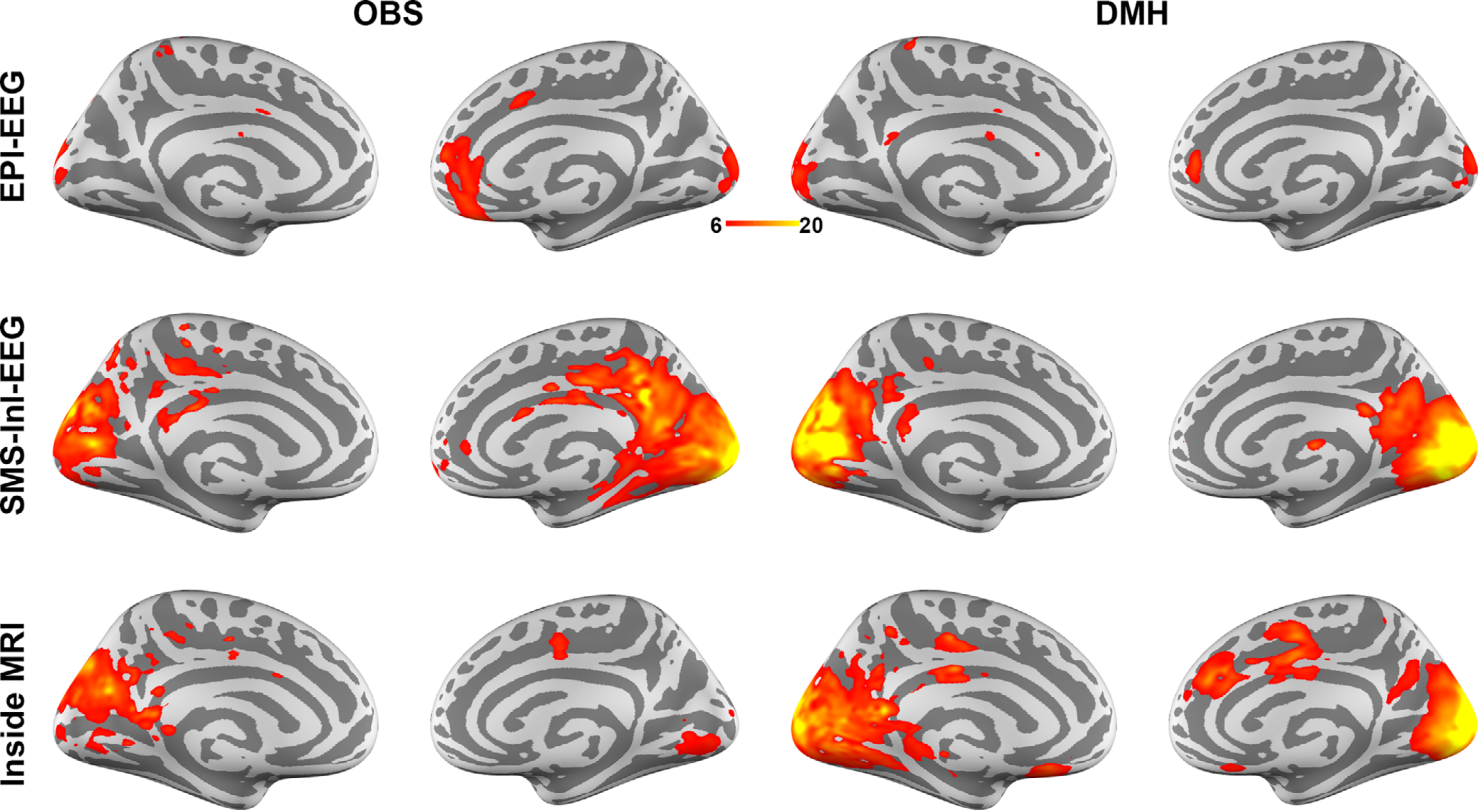
Spatial distributions of 15‐Hz time‐frequency representation of the estimated neuronal current power over cortical surfaces. Distributions were Z scores averaged over the time interval from +200 to +1000 ms after the visual stimulus onset, with respect to the baseline pre‐stimulus interval.

The neuronal current distributions of the 15‐Hz SSVEP were examined next. Figure 4 shows the average (calculated over the time window from +200 to +1000 ms) ratio of the power of the 15‐Hz time‐frequency representation with respect to that in the pre‐stimulus interval. The difference between OBS and DMH was not readily apparent in the EPI‐EEG maps. Both maps showed the expected high power ratios around the posterior occipital lobe with intriguing signals around the right frontal pole. For SMS‐InI‐EEG, the difference between OBS and DMH was clear: a much stronger power ratio of 15‐Hz oscillation was found around the visual cortex in DMH than OBS. This performance was similarly observed for the Inside‐MRI results. The DMH method yielded a stronger and more balanced 15‐Hz power ratio between left and right visual cortices than the OBS method. A strong power ratio around the cingulate cortex was found in both OBS and DMH. This may be due to the supplementary eye field activity to suppress saccades to fixate at the center of the visual field and ignore the flickering visual stimuli at the periphery of the visual field (Schlag‐Rey et al., 1997).

**FIGURE 5:**
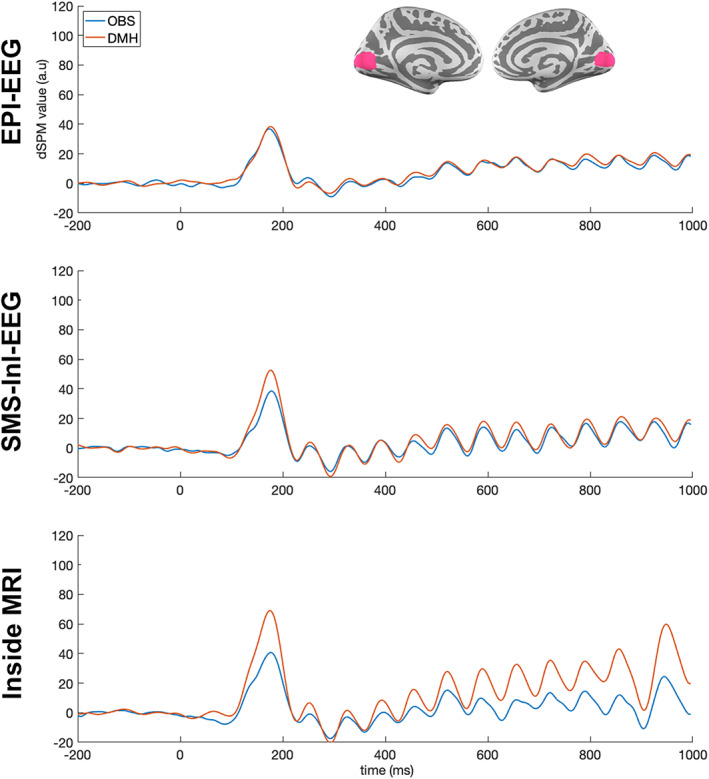
Time courses of the estimated neuronal current over cortical surfaces at the visual cortex with DMH or OBS to suppress BCG artifacts. The boundary of the visual cortex is illustrated by pink cortical patches in the inlet of the top panel.

Figure 5 shows the time courses of the estimated neuronal currents at the visual cortex in three conditions after either OBS or DMH suppressing BCG artifacts. All time courses exhibited a transient response at around 200 ms, followed by oscillatory activities. In EPI‐EEG, the transient response and the following oscillatory activities were visually similar between DMH and OBS. In SMS‐InI‐EEG, a stronger transient response was found with DMH than OBS (56 for DMH and 41 for OBS; 36% gain). Visually clear 15‐Hz oscillation was found in data processed with both methods. DMH visually outperformed OBS in Inside‐MRI by a much stronger transient response (105 for DMH and 56 for OBS; 88% gain) and 15‐Hz oscillation (see quantification next).

**FIGURE 6:**
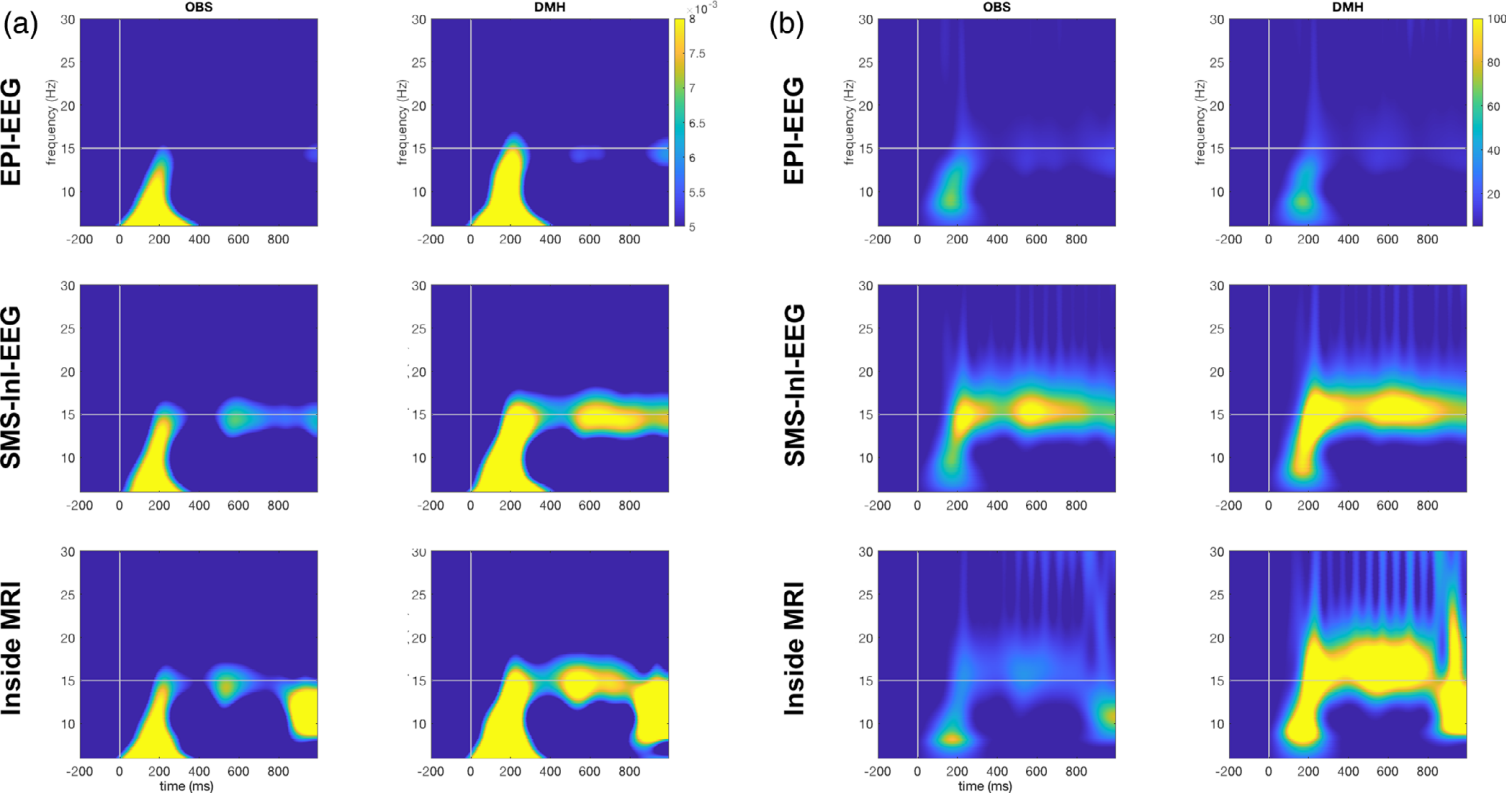
Time‐frequency representations of the estimated neuronal current at the visual cortex using DMH or OBS to suppress BCG artifacts. (a) Plots of time‐frequency representations between 5 Hz and 30 Hz. (b) Plots of noise‐normalized time‐frequency representations between 5 Hz and 30 Hz

Figure 6a shows the TFRs of the estimated neuronal current at the visual cortex. Slightly stronger 15‐Hz oscillation was inspected in EPI‐EEG using DMH than OBS. The 15‐Hz oscillation differences were visually clearer in SMS‐InI‐EEG and Inside MRI conditions. The 15‐Hz oscillation between +0.2 and + 1.0 s after the stimulus onset for DMH and OBS were 4.99 × 10^−3^ ± 8.16 × 10^−4^ and 3.88 × 10^−3^ ± 7.68 × 10^−4^, respectively. DMH offered significantly stronger 15‐Hz oscillation (29% gain; two‐sample *t*‐test *t* = 19.83; *p* < 1 × 10^−20^). In SMS‐InI‐EEG, the 15‐Hz oscillation between +0.2 and + 1.0 s after the stimulus onset for DMH and OBS were 7.57 × 10^−3^ ± 7.24 × 10^−4^ and 5.79 × 10^−3^ ± 6.35 × 10^−4^, respectively. DMH offered significantly stronger 15‐Hz oscillation (31% gain; two‐sample *t*‐test *t* = 36.86; *p* < 1 × 10^−20^). In Inside‐MRI, the 15‐Hz oscillation between +0.2 and + 1.0 s after the stimulus onset for DMH and OBS were 7.62 × 10^−3^ ± 7.24 × 10^−4^ and 5.79 × 10^−3^ ± 5.88 × 10^−4^, respectively. DMH also offered significantly stronger 15‐Hz oscillation (32% gain; two‐sample *t*‐test *t* = 39.09; *p* < 1 × 10^−20^);

We also calculated noise‐normalized TFRs for each data set, where the noise level was separately estimated by the standard deviation of signals in the pre‐stimulus interval at each frequency (Figure 6b). Ratios were taken between post‐stimulus instants and the estimated noise level. Results with DMH and OBS were similar in the time‐frequency representation between 5 Hz and 30 Hz with similar 15‐Hz dynamics in EPI‐EEG. The average 15‐Hz noise‐normalized TFR was 11.07 +/− 3.81 and 9.81 +/− 3.51 for OBS and DMH, respectively. The difference was not significant. DMH shows stronger and persistent noise‐normalized 15‐Hz oscillation than OBS in SMS‐InI‐EEG. Quantitatively, the average 15‐Hz TFR was 83.94 +/− 14.52 and 103.59 +/− 21.72 for OBS and DMH, respectively. The difference between the two methods was significant (two‐sample *t*‐test; *p* < .001). This difference was significant for the EEG data recorded during the Inside‐MRI condition, where the strongest noise‐normalized 15‐Hz oscillation was found. In this case, the noise‐normalized 15‐Hz TFR was 25.16 +/− 5.36 and 111.12 +/− 23.05 for OBS and DMH, respectively (two‐sample *t*‐test; *p* < .0001). Note that the noise‐normalized 15‐Hz oscillation for inside‐MRI was smaller than SMS‐InI‐EEG when using OBS (left middle and bottom panels; Figure 6b). This was likely attributed to the difference in baseline noise level between SMS‐InI‐EEG and Inside‐MRI, because of their similar strength without noise normalization (left middle and bottom panels; Figure 6a).

Based on the boundary of the primary visual cortex as the ground truth for the activated brain areas in the SSVEP experiment from an independent structural MRI and fMRI studies (Benson et al., 2014; Hinds et al., 2008), Figure 7 shows ROC curves used to quantify the sensitivity and specificity of detecting the estimated 15‐Hz neuronal current oscillation in the visual cortex using either OBS or DMH methods in EEG source modeling. For EPI‐EEG, the area under the ROC curve (AUC) value for OBS and DMH was 0.85 and 0.87, respectively. For SMS‐EPI‐EEG, the AUC value for OBS and DMH was 0.93 and 0.98, respectively. For Inside‐MRI, the AUC value for OBS and DMH was 0.91 and 0.97, respectively. The AUC increased progressively from EPI‐EEG, SMS‐InI‐EEG, and Inside‐MRI. In all three conditions, DMH consistently had a larger AUC value than OBS. The bootstrap analysis revealed that the difference in the AUC between DMH and OBS approaches was statistically significant in EPI (*t* = 3.32, *p* = 1.6 × 10^−3^), SMS‐InI (*t* = 3.92, *p* = 2.4 × 10^−4^), and Inside‐MRI (*t* = 10.27, *p* = 1.1 × 10^−14^).

**FIGURE 7:**
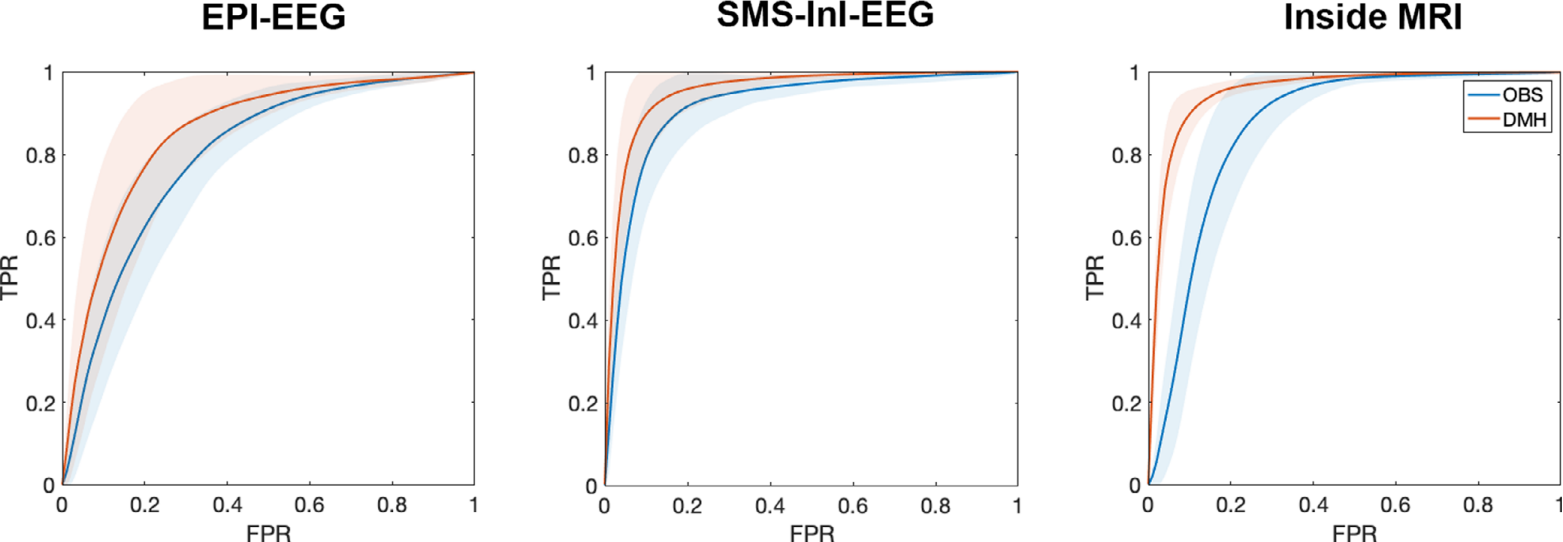
Receiver‐operating characteristic curves of the 15‐Hz time‐frequency representation averaged between 200 and 1000 ms after the visual stimulus onset. SSVEPs with DMH have larger areas under the ROC curves in EPI‐EEG, SMS‐InI‐EEG, and inside‐MRI conditions than OBS. Shaded areas represent one standard error in the bootstrap analysis above and below the ROC from the group averages. FPR, False‐positive rate; TPR, True‐positive rate

## 4 DISCUSSION

The present study proposes a method—dynamic modeling of heartbeats (DMH)—for modeling the ballistocardiogram (BCG) based on recorded EKG data, and then subtracting the artifact from EEG data recorded inside an MRI system. Performance of the DMH method was systematically characterized in comparison to the conventional OBS method in EEG BCG artifact suppression for EEG evoked potentials. Specifically, a steady‐state visual evoked response experiment was undertaken to record EEG data inside an MRI system either concurrently with EPI, with fast MRI, or in the static magnetic field without performing MRI. The DMH approach was stable across choices of parameter combinations (Figure 2). Compared to OBS, DMH significantly improved time courses (Figure 3a) and their 15‐Hz oscillations (Figure 3b) from occipital electrodes over the scalp in BCG suppression. Source modeling of SSVEPs revealed a stronger transient and oscillatory neuronal current in the visual cortex with DMH than with OBS (Figures 4 and 5). The estimated 15‐Hz oscillation in the visual cortex was found to be about 30% stronger with DMH than with OBS in SMS‐InI‐EEG and Inside‐MRI conditions, respectively (Figure 6). An ROC analysis showed that use of DMH resulted in higher power in detecting the oscillatory visual cortex neuronal activity in all three EEG acquisition scenarios (Figure 7). In summary, the proposed DMH method reduced BCG artifacts further than was possible with the OBS method in the experimental conditions that were assessed, resulting in EEG signal time courses that were more related to neuronal activity.

The DMH method likely outperformed OBS in BCG artifact suppression because DMH adaptively identified BCG artifacts by pooling EEG recordings with similar cardiac dynamics, while accounting for variability in cardiac phase and signal amplitudes within the pool. The DMH method assumes that time instants of similar EKG dynamics have similar EEG BCG artifacts. This assumption was supported by early studies on the origin of BCG artifacts induced by the cardiac‐related motion of the head and electrodes in a static magnetic field (Allen et al., 1998; Debener et al., 2008). One limitation of DMH is that the method requires identification of EKG events correlated to EEG signals. If the neuronal responses of interest are asynchronous with cardiac cycles, then DMH is expected to reveal these responses by adaptively synthesizing BCG artifacts from the EEG data with similar cardiac dynamics. The other limitation of DMH is the need to record the EKG, which is used to identify cardiac phase in modeling the BCG artifact. The EKG recording is also required by OBS, but not by the harmonic regression method (Krishnaswamy et al., 2016).

The performance difference between DMH and OBS became progressively smaller from Inside‐MRI, SMS‐InI‐EEG, and EPI‐EEG. This is likely due to the residuals in GA suppression, which is the least and largest in Inside‐MRI and EPI‐EEG, respectively. A better performance by DMH than OBS was consistently observed in evoked responses. Therefore, it is recommended that DMH should be used to process EEG data with MRI collected using a minimal MRI acquisition time and an acceptable spatiotemporal resolution and a field‐of‐view to obtain high‐quality EEG and MRI at the same time.

The DMH method is computationally efficient: it took less than 10 s to complete the BCG suppression on 32‐channel EEG data recorded at 5,000 Hz for approximately 8 min. The calculation was performed by a CPU without the need for dedicated parallel processors or large memory capacity. In comparison, OBS took about 70 s to complete BCG suppression for the same data set using the same computational resource. About 7‐fold higher computational load in OBS than DMH is due to the use of Principal Component Analysis in OBS but not in DMH. Higher computational demand is common across component analysis methods (Debener et al., 2008; Nakamura et al., 2006; Niazy et al., 2005b; Srivastava et al., 2005).

In the present study, OBS was taken as the benchmark to test DMH performance. Other alternatives for advanced BCG suppression include harmonic regression (Krishnaswamy et al., 2016) and deep learning strategies such as BCGnet (McIntosh et al., 2021). However, harmonic regression gave an evoked response similar to OBS processing for EEG recorded inside a 3 T magnet in the absence of MRI, an ideal case of avoiding potential residual errors that arise from GAs during EEG‐MRI (Krishnaswamy et al., 2016). Given concurrently recorded EPI and EEG data inside a 3 T MRI system, the BCGnet approach and OBS generated similar evoked responses even though the response variability was smaller using BCGnet than OBS (McIntosh et al., 2021). In contrast, in the present work, DMH gave more specific evoked responses. Given the preliminary nature of these results, it would be useful to conduct additional comparisons of DMH, OBS, and BCGnet in other EEG‐MRI scenarios in the near future.

Like OBS, DMH depends on the availability of the EKG to estimate time instants in terms of phases of cardiac cycles. Therefore, the quality of EKG affects the performance of DMH. In cases of low‐quality EKG and associated failures to detect QRS complexes, DMH may not perform optimally. Component analysis methods (Debener et al., 2008; Nakamura et al., 2006; Niazy et al., 2005b; Srivastava et al., 2005), however, assume either statistical independence or uncorrelation (orthogonality) between signal and noise time series to separate neuronal activity from measurement artifacts. The DMH method does not rely on these assumptions, nor does it require substantial ancillary hardware to record signals specific to noise processes (Chowdhury et al., 2014; Mullinger, Castellone, & Bowtell, 2013; Mullinger, Havenhand, & Bowtell, 2013). A typical setup of MRI‐compatible EEG with EKG suffices to suppress BCG noise and recover neuronal signals.

The present study investigated EEG evoked potentials corrupted by BCG inside a 3 T MRI scanner. The BCG artifact has been shown to scale with MRI field strength (Debener et al., 2008; Mullinger, Castellone, & Bowtell, 2013; Mullinger, Havenhand, & Bowtell, 2013), suggesting that EEG data recorded in 7 T MRI systems may have higher quality after DMH processing than OBS processing. In addition to improving the EEG‐MRI data from healthy participants, DMH may also be useful to assist clinicians in detecting interictal spikes (IIS) from concurrent EEG‐fMRI of epilepsy patients. In such clinical applications, detecting IIS occurrences is the crucial step for subsequent analysis of fMRI data. Improving the EEG quality may improve the sensitivity and specificity of spike detection and subsequent fMRI‐based delineation of the irritative zones, thus assisting neurosurgical decision‐making.

The present study demonstrated the performance of DMH in EEG evoked potentials experiments. However, DMH can be helpful beyond this exemplary case. Recently, converging evidence suggested that the human brain has evolved to deal with complex naturalistic, which can elicit neuronal responses more reliably than simplified stimuli in conventional laboratory experiments (Belitski et al., 2008; Mechler et al., 1998; Yao et al., 2007). Accordingly, there is an emerging trend of using naturalistic stimuli, including movies, TV shows, and musical pieces, to study human brain function. (For review, see [Hasson et al., 2010]). This experimental technique has been suggested to be more appropriate to probe the neuronal responses related to complex cognitive processes common in our daily life, such as narrative comprehension (Regev et al., 2013; Wilson et al., 2008) and movie watching (Hasson et al., 2004; Hasson et al., 2008; Jaaskelainen et al., 2008). To date, most neuroimaging studies using complex naturalistic stimuli are limited to fMRI. Concurrent EEG‐MRI study with the complex naturalistic stimuli paradigm can elucidate the neurophysiological basis and the neurovascular coupling in an ecologically valid setting. DMH will be useful in such studies because the improved EEG quality can disclose neuronal responses more sensitively by suppressing BCG artifacts.

In clinical neurology, integrating EEG and fMRI is one useful way to localize epileptiform activity generators (Gotman et al., 2004; Ives et al., 1993; Krakow et al., 1999; Seeck et al., 1998). Specifically, the locations of IIS generators have been estimated by identifying brain areas with significant hemodynamic changes after inter‐ictal spikes (IIS). The IIS time window can either be annotated by epileptologists (Gotman, 2008; Lemieux et al., 2001; Salek‐Haddadi et al., 2006) or informed by the correlation between the instantaneous EEG topography and patient‐specific EEG topographies (Grouiller et al., 2011). Full concordance between fMRI maximum response and surgical resection is indicative of seizure freedom, whereas a resection leaving the fMRI maximum response intact is likely to lead to a poor outcome (An et al., 2013). We plan to further quantify the DMH performance in spontaneous neuronal activity, including epileptic events and spontaneous oscillations, to translate this method to clinical practice service.

## AUTHOR CONTRIBUTIONS

Hsin‐Ju Lee, Wen‐Jui Kuo, Simon J Graham, and Fa‐Hsuan Lindesigned the experiment. Hsin‐Ju Leeand Fa‐Hsuan Lin developed the method. Hsin‐Ju Lee collected the data. Hsin‐Ju Lee and Fa‐Hsuan Lin analyzed the data. All authors wrote the manuscript.

## Data Availability

The data that support the findings of this study are available on request from the corresponding author. The data are not publicly available due to privacy or ethical restrictions.
